# Remote and automated high-throughput powder diffraction measurements enabled by a robotic sample changer at SSRL beamline 2-1

**DOI:** 10.1107/S1600576723007148

**Published:** 2023-09-20

**Authors:** Kevin H. Stone, Monty R. Cosby, Nicholas A. Strange, Vivek Thampy, Richard C. Walroth, Charles Troxel Jr

**Affiliations:** a Stanford Synchrotron Radiation Lightsource, SLAC National Accelerator Laboratory, Menlo Park, CA 94025, USA; Australian Synchrotron, ANSTO, Australia

**Keywords:** powder diffraction, synchrotron beamlines, automated measurement, robotic sample changers

## Abstract

The general-purpose powder diffractometer beamline (BL2-1) at the Stanford Synchrotron Radiation Lightsource (SSRL) is described. The evolution of design and performance of BL2-1 are presented, in addition to current operating specifications, applications and measurement capabilities. Recent developments involve a robotic sample changer enabling high-throughput X-ray diffraction measurements, applicable to mail-in and remote operations. *In situ* and *operando* capabilities to measure samples with different form factors (*e.g.* capillary, flat plate or thin film, and transmission) and under variable experimental conditions are discussed. Several example datasets and accompanying Rietveld refinements are presented.

## Abstract 2

The general-purpose powder diffraction beamline at Stanford Synchrotron Radiation Lightsource (SSRL) is described. The latest developments at the beamline, including the implementation of a robotic sample changer for mail-in operations, are discussed and several datasets measured from this setup are included for reference.

## 1. Introduction

Powder diffraction is a workhorse technique in physics, chemistry, materials science and a diverse range of other scientific areas. Great progress has been made in the use of laboratory-based instruments for routine and advanced powder diffraction measurements. However, synchrotrons still offer significant advantages and continue to see a large demand for high-quality synchrotron powder diffraction data. The main advantages are the increased speed of measurement, enabling *in situ* and *operando* studies; improved signal-to-noise ratio, enabling the study of low-concentration species; tunable energy, enabling resonant studies or reduced fluorescence background signals; and significantly higher resolution, extending the power of powder diffraction to structure solution and studies of subtle structural changes. Powder diffraction is also one of the more accessible synchrotron-based techniques, making it an excellent introduction for users new to the opportunities afforded by large user facilities. As a result, dedicated powder diffraction beamlines can be found at nearly every large synchrotron and neutron user facility (Hodeau *et al.*, 1998 ▸; Lee *et al.*, 2008 ▸; Abdellatief *et al.*, 2022 ▸; Dippel *et al.*, 2015 ▸; Toraya *et al.*, 1996 ▸; Willmott *et al.*, 2013 ▸; Huq *et al.*, 2011 ▸; Garlea *et al.*, 2010 ▸; Wallwork *et al.*, 2007 ▸; Shi *et al.*, 2013 ▸; Thompson *et al.*, 2009 ▸; Pattison *et al.*, 1989 ▸; Fauth *et al.*, 2013 ▸; Leontowich *et al.*, 2021 ▸; Rebuffi *et al.*, 2014 ▸; Carvalho *et al.*, 2016 ▸). Here, the general-purpose powder diffractometer beamline 2-1 (BL2-1) at the Stanford Synchrotron Radiation Lightsource (SSRL) is described.

## 2. Beamline history and overview

In November 1976, the Stanford Synchrotron Radiation Project (SSRP), the precursor to the Stanford Synchrotron Radiation Lightsource (SSRL), expanded with the opening of Beam Port II, consisting of three operational end stations dedicated to synchrotron X-ray science based around the Stanford Position Electron Asymmetric Ring (SPEAR) accelerator. What would become beamline 2-1 consisted of a double-focusing toroidal mirror and a monochromator of two independent Ge(111) crystals. The beamline operated until 1991 when it was closed to allow staff to better support users on the remaining operational beamlines. In 1996, the beamline saw new life as a dedicated powder diffraction instrument to support the many powder diffraction experiments that had been using the large general-purpose diffractometers at beamlines 7-2 and 10-2, which were both highly oversubscribed and overly complicated for powder diffraction work. Beamline 2-1 was commissioned and opened to general users in 1997 and has been in continuous use since, undergoing numerous upgrades to the optics and in-hutch equipment over that time alongside the accelerator upgrades to the current third-generation source. From the beginning, beamline 2-1 has been envisioned as an instrument to enable routine collection of high-quality high-resolution powder diffraction and reflectivity data without compromising the ability to accommodate highly customized *operando* and *in situ* sample environments.

Today BL2-1 has a 1.5 mrad angular acceptance from a 1.3 T bend magnet from SPEAR3 operating at 3 GeV and 500 mA. This is focused using a cylindrically bent Rh-coated mirror which feeds into a Si(111) double-crystal monochromator supplying X-rays in the energy range of 5500–17500 eV in a spot size of approximately 200 × 500 µm. The beamline is equipped with a two-circle diffractometer based around a Huber 430 and Huber 420 set of rotation stages with two detector positions on the 2θ arm. One detector position is dedicated to a high-resolution setup based around a Si(111) analyzer crystal, with the other detector position suitable for routine reconfiguration. Although the second detector position can be reconfigured, it is typically used with a Pilatus 100K small area detector for rapid data collection at intermediate angular resolution. This configuration is especially suitable for *operando* and *in situ* studies. The X-ray energy at beamline 2-1 is tunable, making it suitable for resonant diffraction studies covering the majority of the first-row transition metal *K* edges and many of the lanthanide *L* edges. The beamline is controlled using the *SPEC* (Certified Scientific Software, Cambridge, MA, USA) interface.

### 2.1. Robotic sample changer

**Figure 1:**
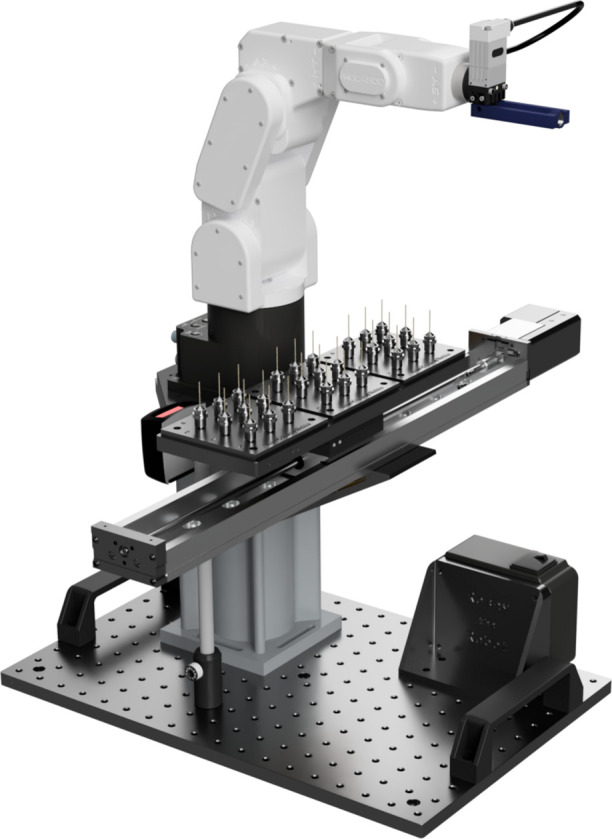
CAD model of the robotic sample changing platform. The robotic sample changer is designed around a Meca500 six-axis robotic arm and mounted along with the sample cassette and barcode scanner on an optical breadboard. The entire platform has dimensions of approximately 450 × 300 × 600 mm (length × width × height) and is designed to be easy to install or remove from the beamline by a single person.

The rapid data collection enabled through use of the Pilatus 100K small area detector, with full data collection times on the order of 1–10 min, has motivated the development of a robotic sample changer. The robotic sample changer can run a large number of samples in a standard capillary geometry in a fully automated and unsupervised mode. This mode of operations allows the beamline to collect data on large numbers of samples very efficiently and enables both mail-in and remote operations. Automating measurements is advantageous in that data acquisition can be standardized and optimized, while mail-in or remote operations allow for a more geographically diverse user base, immunity from travel disruptions, and access for under-represented colleges and universities who traditionally have not taken advantage of synchrotron user facilities. The robotic sample changing platform is a modular design built around a small six-axis robotic arm from Mecademic. It is focused on enabling high-throughput high-quality data collection for routine samples but can be easily installed or removed from the beamline so as to still enable complex *in situ* or *operando* setups to be installed (Fig. 1 ▸).

Powder samples can be loaded into Kapton capillaries of diameter ∼0.7 mm. These in turn are mounted into magnetic steel bases with unique sample ID numbers encoded in barcodes on the bottom. Up to 27 such samples can be mounted onto a sample plate, which in turn mounts to the robotic sample changer with a magnetic kinematic mount as shown in Fig. 1 ▸. The positional reproducibility of the kinematic mounted cassettes enables rapid sample changeover. Each plate can be measured using one of two approaches. In the first, data collection is fully automated using a standard set of scan parameters designed to produce high-quality data for essentially any sample. While the counting time, angular range and resulting resolution from this strategy may be unnecessary for many samples, the speed of measurement means that this costs little. Powder diffraction data collected in this manner for several example materials are included as supplemental materials, along with simple structural refinements to illustrate the data quality achievable through this access model (Coelho, 2018 ▸; Rowles, 2022 ▸). For more complex measurements, *e.g.* resonant diffraction measurements, the second approach uses a graphical user interface (GUI) which allows the user to select a sample to mount using the robot while retaining full control over the beamline remotely.

The ability to measure air-sensitive samples in glass capillaries was recently integrated with the robotic sample changing capabilities at BL2-1. In contrast with conventional approaches for sealing glass capillaries, which typically involve melting paraffin wax, it was recently recognized that commercially available arc lighters can produce a plasma sufficiently hot in a nitrogen environment to melt and seal thin-walled (∼10 µm) borosilicate and soda-lime glass capillaries. The arc lighter also generates extremely localized heating, which is necessary for preventing thermal reactions in sample powders. Caution should be exercised when sealing glass capillaries in an inert atmosphere. An insulating medium (rubber mat, alumina plate) should be used to avoid discharge to the glovebox. Sealing the glass directly through a melt generates a more robust and gas-tight seal than paraffin wax. The sealed glass capillaries can be secured within the Kapton capillaries used for the robotic sample bases or directly inserted into the bases using modeling clay.

#### 2.1.1. Robotic sample changing control GUI

**Figure 2:**
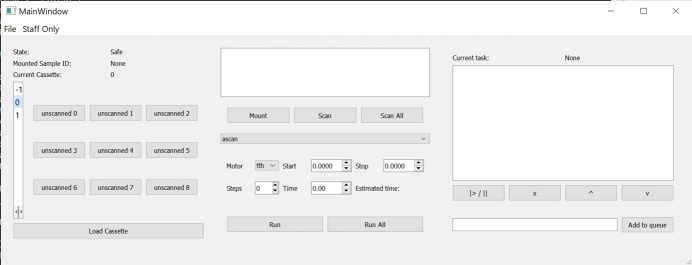
Screenshot of the robotic sample changing control GUI showing the various buttons to choose a sample, mount or unmount that sample, and run simple scans. The task queue is shown empty at the right side of the window.

For complex measurement strategies for remote experiments, the robotic sample changer can be controlled through a GUI which has been developed using Python. This enables the user to select a sample from any of the 27 positions in the sample cassette. Full control over the beamline can be exercised through this interface or in combination with the standard command line *SPEC* interface. The GUI is separated into three main columns (Fig. 2 ▸). The leftmost column allows the user to select the sample; a 3 × 3 grid of samples is displayed with the sample ID shown once the sample barcode has been scanned. The appropriate number of 3 × 3 grids are given with the ability to switch between them. For the current setup, there are three such grids which can be translated between. If sample metadata are available, these can be loaded into the program and displayed in the text box at the center of the GUI. In the center section, basic controls to scan the barcode of a sample, mount and dismount a sample from the diffractometer, and set up simple data collection scans are available. Multiple commands can be queued, which are then shown in the text box at the right of the GUI. Commands in the queue can be rearranged to build up and edit the full experimental pipeline. Arbitrary *SPEC* commands can be entered into the queue using the command line at the bottom right of the interface. This ensures that complex workflows limited only by the beamline itself can be built through this interface.

The robot sample changer GUI is meant to allow either beamline staff or users to operate the beamline fully remotely. Using this interface, they can control the robot to mount any of the available samples onto the diffractometer. Using either the standard scan controls or a series of specific *SPEC* commands, complex workflows can be developed for experiments beyond the simple measurements used in the fully automated operation mode. For example, the sample can be translated to measure at different points, or the energy can be changed for resonant diffraction measurements. The queue can be used to build the workflow for as many of the available samples as desired. In addition to running the beamline remotely, this lets the user set up and run complex experiments without the need for constant interaction with the beamline, improving efficient use of beamtime.

### 2.2. *Operando* and *in situ* chamber designs

In addition to the robotic sample changer and the standard capillary geometry for powder diffraction measurements, the beamline is routinely reconfigured for a number of *operando* and *in situ* sample environments. While many of these are maintained by SSRL and available to general users, custom setups unique to particular user groups are also supported. For capillary geometry measurements, temperature control can be achieved through the use of an Oxford cryostream for low-temperature measurements down to approximately 100 K or through the use of a capillary heater with gas control based around the design of Chupas *et al.* (2008 ▸) as developed by Hoffman *et al.* (2018 ▸).

SSRL beamline 2-1 also supports a number of flat-plate and thin-film sample capabilities, including the ability to measure X-ray reflectivity. The most standard of these is a helium chamber for *ex situ* measurements under an inert environment. Measurements at elevated temperatures can be performed using either a custom-designed heating chamber (up to 500°C), an Anton Paar DHS 900 (up to 900°C) or an Anton Paar HTK1200N (up to 1200°C). These are compatible with bulk powder samples loaded into single-crystal zero-background plates or, more commonly, thin-film/flat-plate samples. These can be measured at a symmetric quasi Bragg–Brentano geometry or in a grazing-incidence configuration. All basic temperature control setups are standard configurations available through the general user program at SSRL.

Many user groups have developed more customized sample environments for particular experiments. Several of these were developed by or in collaboration with beamline staff and can be made available to users through collaboration with beamline personnel. Among these are a solar *operando* chamber which enables illumination of the sample by a solar simulator and current–voltage measurement through a Keithley DC power supply (Schelhas *et al.*, 2016 ▸). Atmospheric control as well as moderate temperature control is possible with this setup. Several electrochemical cells have been developed for use at the beamline as well. One configuration which has been modified for a number of use cases was developed for *operando* reflectivity measurements during electrochemical lithiation of silicon (Cao *et al.*, 2016 ▸). This basic design has found use for a number of similar electrochemical studies of flat surfaces with both reflectivity and grazing-incidence diffraction measurements.

## 3. Beamline performance

The general-purpose nature of the beamline means that the performance is often dependent on the experimental configuration being used. For the majority of measurements, this involves the use of the Pilatus 100K area detector at a sample-to-detector distance of 700 mm. At this distance the detector covers slightly more than 5° in 2θ in a single image. With a fixed detector position, it is possible to monitor a small angular range with sub-second time resolution for quickly changing samples. Images can also be merged together for larger angular coverage. By using an area detector, only a small number of images are needed to provide full coverage over large angular ranges up to 120° in 2θ. Overlap in subsequent images can prevent any gaps in the data as well as improving the measured signal by averaging multiple measurements of the same angle over multiple detector frames. With this approach, powder diffraction covering the full angular range from 0 to 120° can be made with as few as 24 images, taking as little as 1 min for the full measurement. Typically, smaller steps resulting in greater overlap between images are used so that on the order of 100 images are used, taking a few minutes to collect using a variable count time strategy (David, 2004 ▸).

**Figure 3:**
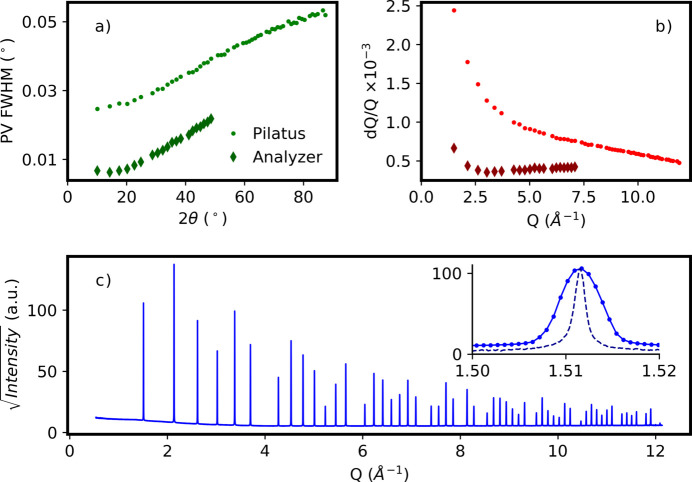
Resolution performance for capillary samples using the Pilatus 100K small area detector and compared with that obtained with an analyzer crystal. Data were collected for a sample of NIST SRM 660c LaB_6_ loaded into a 0.7 mm-diameter Kapton capillary and measured with an X-ray energy of 17 keV. Peak widths obtained by fitting individual peaks to a pseudo-Voigt (PV) profile (*a*) and resolution d*Q*/*Q* obtained for those peak widths (*b*) are shown for data collected with the Pilatus small area detector (small circles) and the analyzer crystal setup (diamonds). The full pattern, plotted as the square root of the measured intensity versus *Q*, obtained from this measurement is shown in (*c*), with the 111 peak shown in the inset for both Pilatus (solid line) and analyzer (dashed line) setups.

By the nature of using an area detector, the beamline resolution is a strong function of the measurement geometry. This is especially true for grazing-incidence measurements where the beam size and incidence angle determine the footprint of the X-ray beam on the sample which is then projected onto the detector. For capillary measurements, the capillary diameter plays a strong role in the instrumental resolution in a similar manner, especially at higher scattering angles. The typical instrumental resolution for a 0.7 mm-diameter capillary, which is standard for the robotic sample changer, is shown in Fig. 3 ▸. A sample of NIST SRM 660c lanthanum hexaboride was measured in a nominally 0.7 mm Kapton capillary and the peaks were fitted using a pseudo-Voigt peak profile. This sample shows minimal peak broadening due to crystallite size or microstrain. Although higher resolution and reduced background can be obtained by using an analyzer crystal, as seen in Fig. 3 ▸, the data quality available in the configuration with the area detector is sufficient for most experiments. Several example datasets collected with this configuration including simple Rietveld refinements are provided in the supplemental materials (see Table S1). The measurement speed is also significantly faster than that which is possible with an analyzer crystal, even with the massively parallel analyzer crystal setups available at several other synchrotron facilities (Hodeau *et al.*, 1998 ▸; Lee *et al.*, 2008 ▸; Thompson *et al.*, 2009 ▸; Fitch & Dejoie, 2021 ▸). For standard capillary measurements, throughputs of >100 samples per day are easily achieved, and for *in situ* studies time resolutions of seconds to minutes are routine.

## 4. Conclusions and future developments

Here we have described the latest developments at the general-purpose powder diffraction instrument at SSRL beamline 2-1. The development of a modular robotic sample changing platform has enabled high-throughput high-resolution powder diffraction consistent with both a mail-in sample program and fully remote operations. The standardization of *ex situ* powder diffraction measurements for bulk samples is complemented by the routine reconfiguration of the sample environment to accommodate complex *operando* and *in situ* experimental platforms. This focus on non-standard sample environments and beamline configurations has been a point of emphasis since the inception of this beamline.

Looking forward, we anticipate a greater emphasis on high-throughput standard measurements. This standardization will be extended to non-ambient conditions, especially temperature-dependent measurements, to make performing these complex measurements as simple and user friendly as possible. The reason for this is twofold. First, by standardizing as many of the standard measurements as possible, we ensure that the configuration is optimized to produce the highest-quality data rather than trying to support multiple similar but distinct sample environments. Second, this reduces the level of effort needed to set up and support these configurations, which in turn enables staff to provide a higher level of support and focus on developing new capabilities driven by the demands of the user community. We anticipate an increasing demand for both standard *ex situ* and custom *in situ* measurements and expect this approach to provide the best balance of support for both types of experiment. By moving towards more standardized setups for as many of the commonly used configurations as possible, we can also extend access to high-quality synchrotron data through remote operations to user groups who have traditionally not used synchrotron instrumentation.

## Supplementary Material

Collection of example datasets including refinements. DOI: 10.1107/S1600576723007148/vb5062sup1.zip

Supporting information file. DOI: 10.1107/S1600576723007148/vb5062sup2.pdf
