# Supplementary material for: Remote and automated high-throughput powder diffraction measurements enabled by a robotic sample changer at SSRL beamline 2-1
Source: J Appl Crystallogr. 2023 Sep 20;56(Pt 5):1480–4. doi: 10.1107/S1600576723007148 (PMC10543666; doi:10.1107/S1600576723007148)
Supplement: Supplementary file 2 [file j-56-01480-sup2.pdf]

JOURNAL OF APPLIED CRYSTALLOGRAPHY

Volume 56 (2023)

Supporting information for article:

Remote and Automated High Throughput Powder Diffraction Measurements Enabled by a Robotic Sample Changer at SSRL Beamline 2-1

Kevin H. Stone, Monty Cosby, Nicholas A. Strange, Vivek Thampy, Richard C. Walroth and Charles Troxel Jr

Table S1

Table S2 List of example data sets measured using the automated data collection strategy and the robotic sample changer:

**Sample ID**	**Sample Composition**	**Sample Notes**	**Refinement Included**	**Refinement Notes**
0001	LaB_6	NIST SRM 660c, lanthanum hexaboride	TA, GSASII	Includes instrument parameter file for GSAS-II created from Pawley refinement.
0002	SiO_2	NIST SRM 1878b, alpha-quartz (respirable)	TA	Includes Pawley fit in TOPAS-Academic.
0003	ZnS	Zinc Sulfide phosphor material	TA	Includes individual peak fit, indexing, and Pawley fit in TOPAS-Academic.
0047	$Pt(O_2C_5H_7)_2$	Platinum(II) bis(acetylacetonate) diluted with diamond powder	TA	Includes individual peak fit, indexing, and Pawley fit in TOPAS-Academic.
0093	Re	Rhenium metal diluted with diamond powder	TA	Includes individual peak fit, indexing, and Pawley fit in TOPAS-Academic. Cylindrical absorption correction included to keep thermal parameters physical.
0188	Er_2O_3	Erbium (III) Oxide mixed with diamond powder to dilute	TA	
0911	Li_2CO_3	Lithium Carbonate - Sigma Aldrich item 255823-100G	TA	Includes individual peak fit, indexing, and Pawley fit in TOPAS-Academic.
0912	MnO	Manganese(II) Oxide 60 mesh - Sigma Aldrich item 377201-500G	TA	Includes individual peak fit, indexing, and Pawley fit in TOPAS-Academic. Correct indexing is 14th best solution found. Cylindrical absorption correction included to keep thermal parameters physical.

0913	Mn_2O_3	Manganese (III) Oxide - Sigma Aldrich item 463701-25G	TA	Rietveld refinement suggests minor impurities of Mn_3O_4 and β-MnO_2.
0916	Fe_2O_3	Iron(III) Oxide - Sigma Aldrich item 529311-5G	TA	Rietveld refinement suggests minor impurity of FeO(OH).
0919	Sb_2O_3	Antimony (III) Oxide - Sigma Aldrich item 379255-50G	TA	Includes individual peak fit, indexing, and Pawley fit in TOPAS-Academic.
0927	TiO_2	TiO_2 less than 100nm particle size, mixture of Anatase and Rutile - Sigma Aldrich	TA	Would likely benefit from a more advanced size and strain model for peak broadening.
0933	Cr_2O_3	Chromium (III) Oxide - Sigma Aldrich item 393703-100G, sample is hygroscopic and bottle was freshly opened just prior to loading the sample into the capillary and measuring on July 10, 2022	TA	

Rietveld refinements were done in TOPAS-Academic v6 (unless otherwise noted) and output generated using the pdCIF macros developed by Matthew Rowles[19]. Refinement data are plotted using the pdCIFplotter program[20]. The included refinements are not meant to be considered as final, these have been refined to basic structural models to give an overall impression of the data quality. Further improvements to the Rietveld refinement models are very possible. Similarly, the CIF output is not intended to be comprehensive, but of sufficient detail to enable easy plotting of the refinement result and the resulting structures. Plots of the Rietveld refinements performed in TOPAS-Academic are shown in Figures S1-S13.

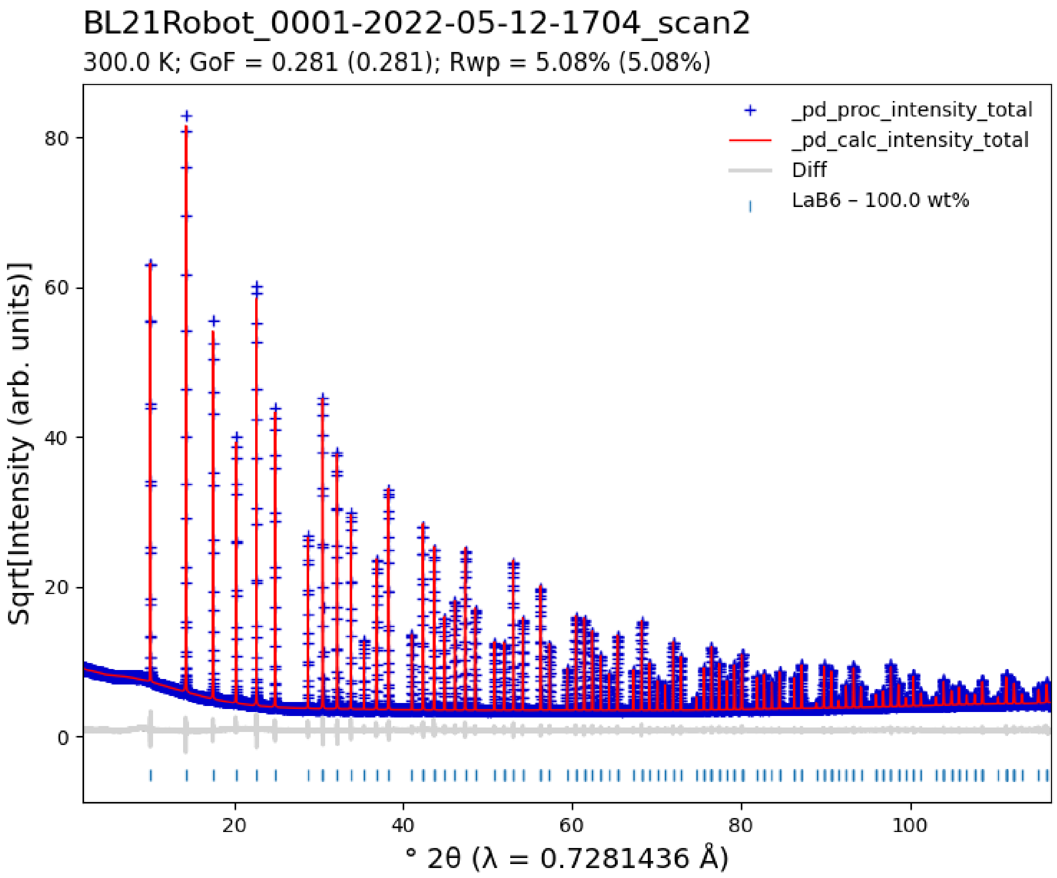

Figure S1 Rietveld refined data for sample BL21Robot_0001, lanthanum hexaboride powder. Measured data are shown as blue crosses, calculated pattern in red with the difference shown below in grey. Tick marks indicate allowed peak positions.

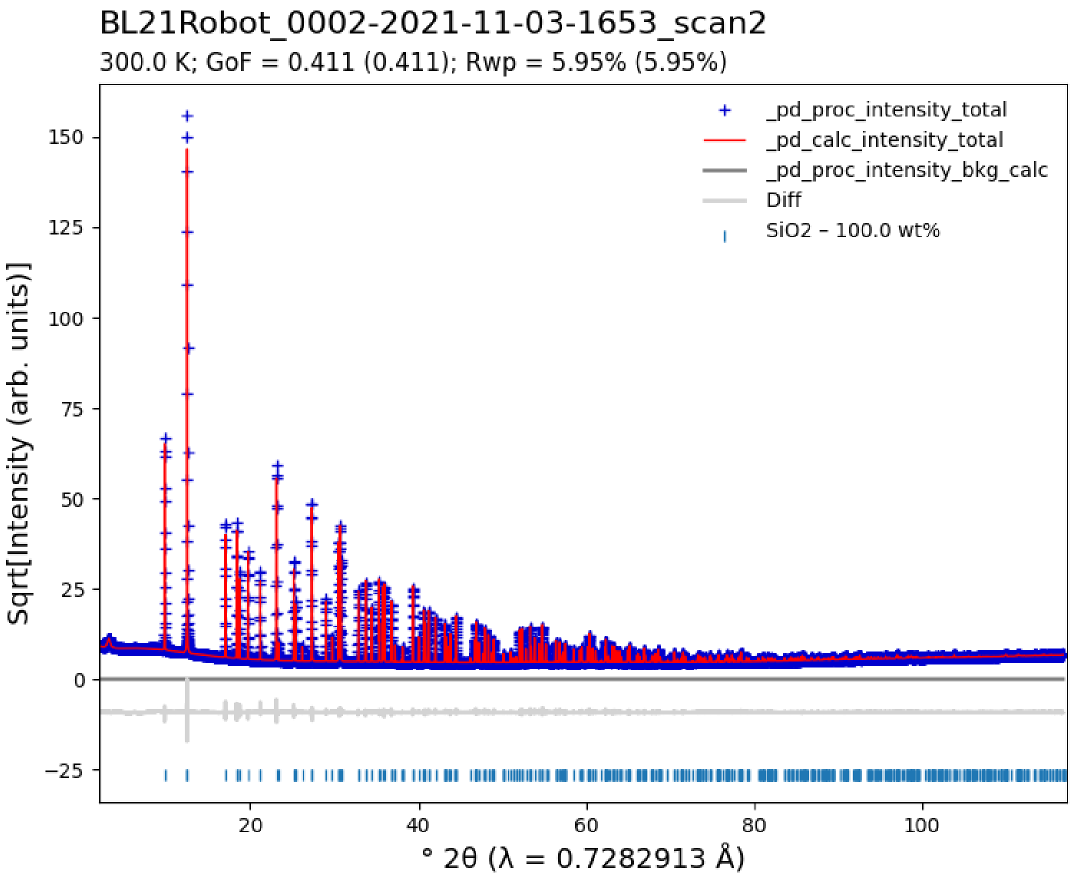

Figure S2 Rietveld refined data for sample BL21Robot_0002, α-quartz. Measured data are shown as blue crosses, calculated pattern in red with the difference shown below in grey. Tick marks indicate allowed peak positions.

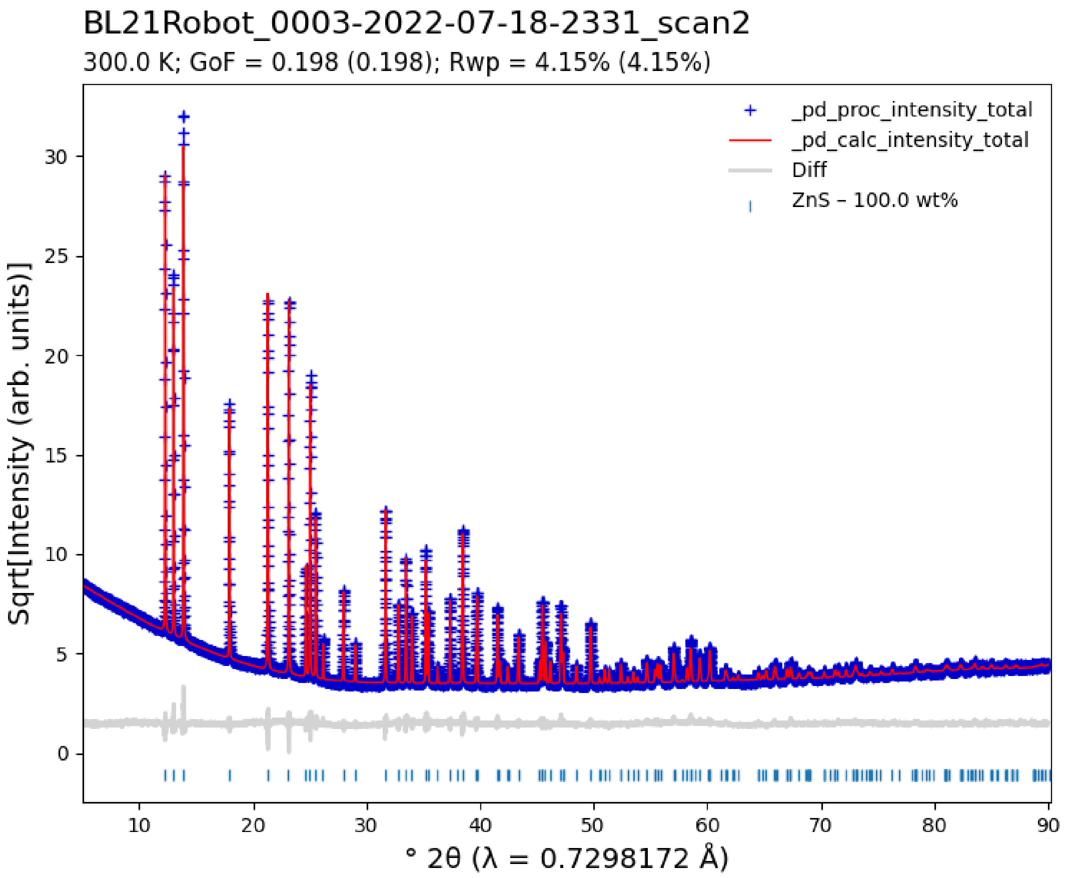

Figure S3 Rietveld refined data for sample BL21Robot_0003, zinc sulfide phosphor powder. Measured data are shown as blue crosses, calculated pattern in red with the difference shown below in grey. Tick marks indicate allowed peak positions.

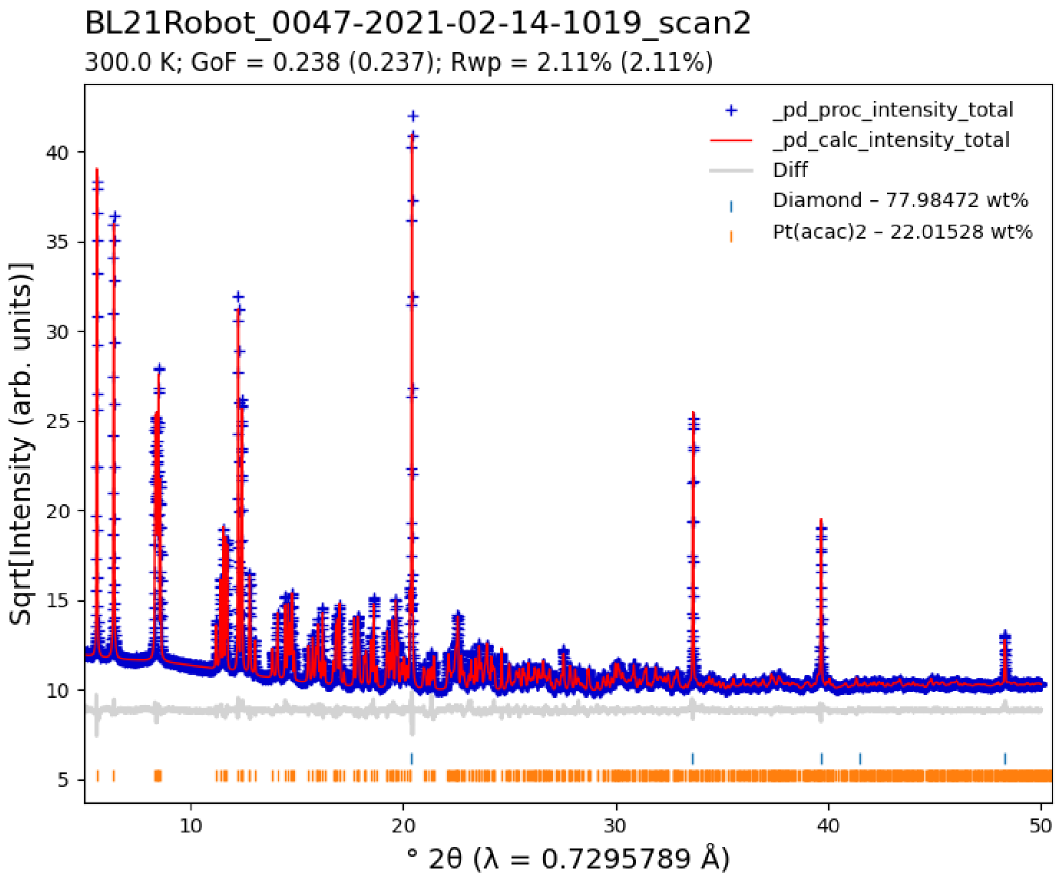

Figure S4 Rietveld refined data for sample BL21Robot_0047, platinum (II) bis(acetylacetonate) diluted with diamond powder. Measured data are shown as blue crosses, calculated pattern in red with the difference shown below in grey. Tick marks indicate allowed peak positions.

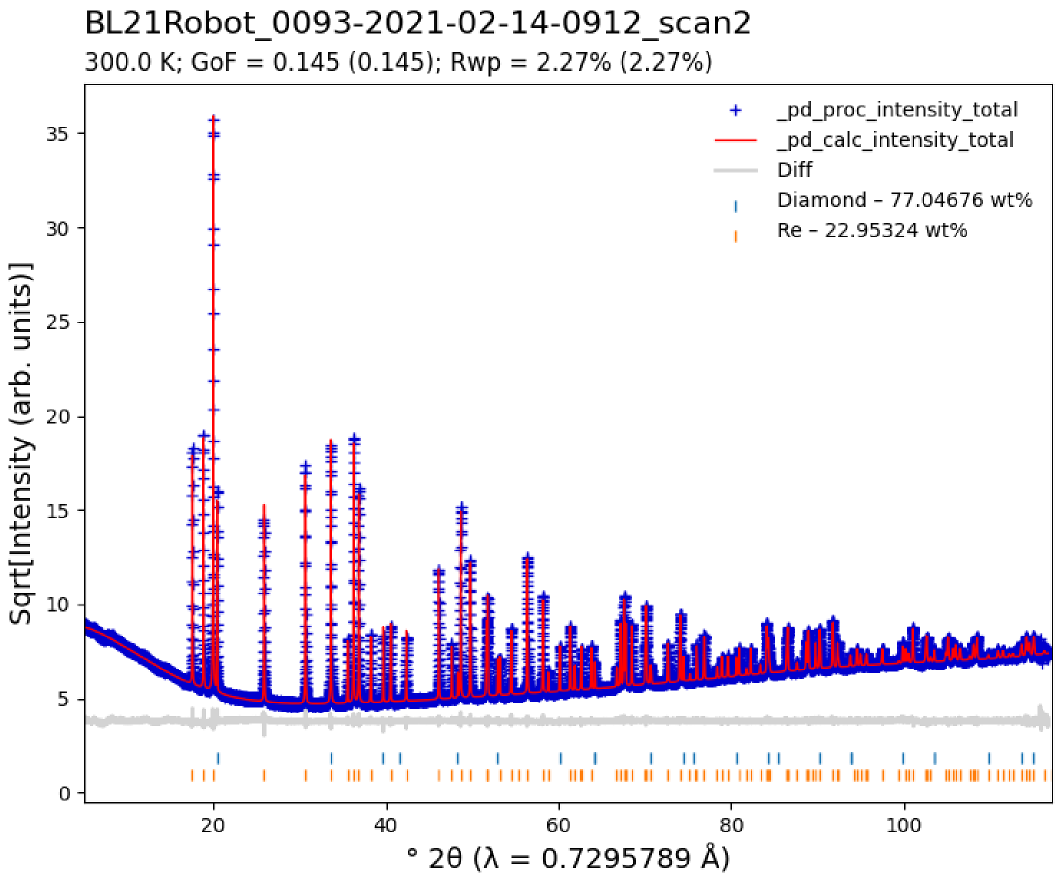

Figure S5 Rietveld refined data for sample BL21Robot_0093, rhenium metal diluted with diamond powder. Measured data are shown as blue crosses, calculated pattern in red with the difference shown below in grey. Tick marks indicate allowed peak positions.

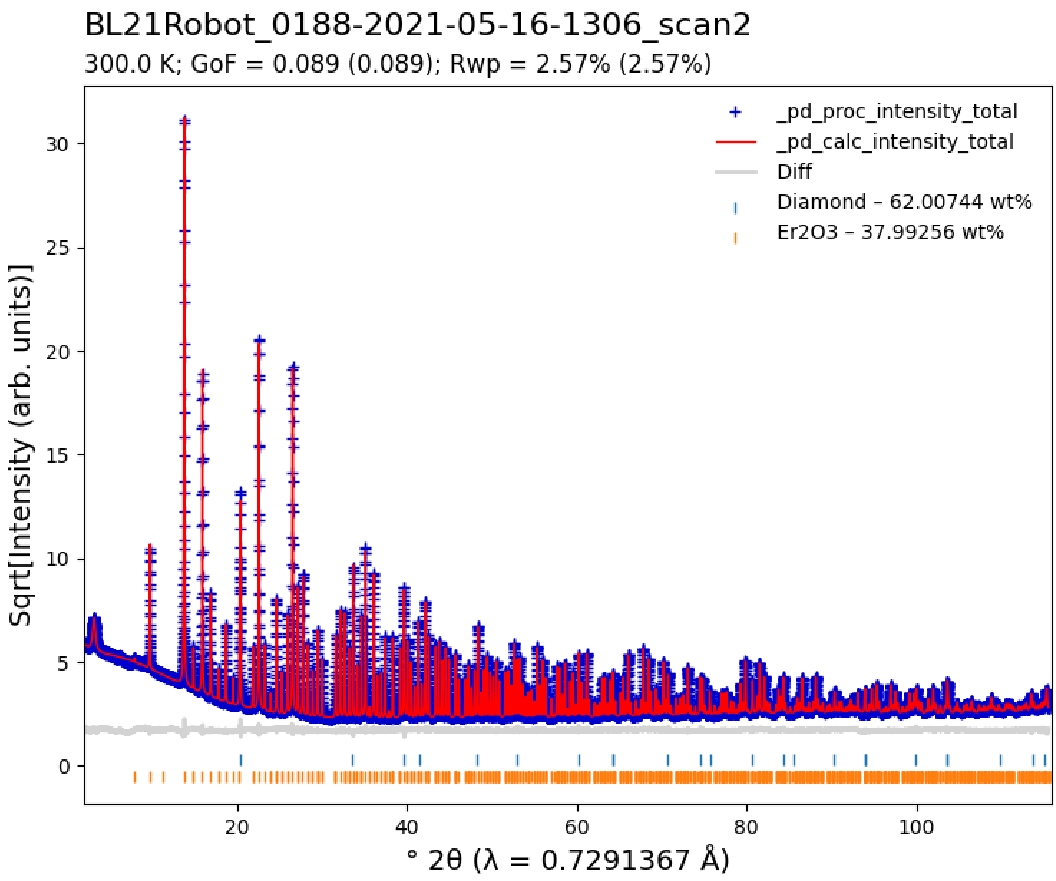

Figure S6 Rietveld refined data for sample BL21Robot_0188, erbium (III) oxide diluted with diamond powder. Measured data are shown as blue crosses, calculated pattern in red with the difference shown below in grey. Tick marks indicate allowed peak positions.

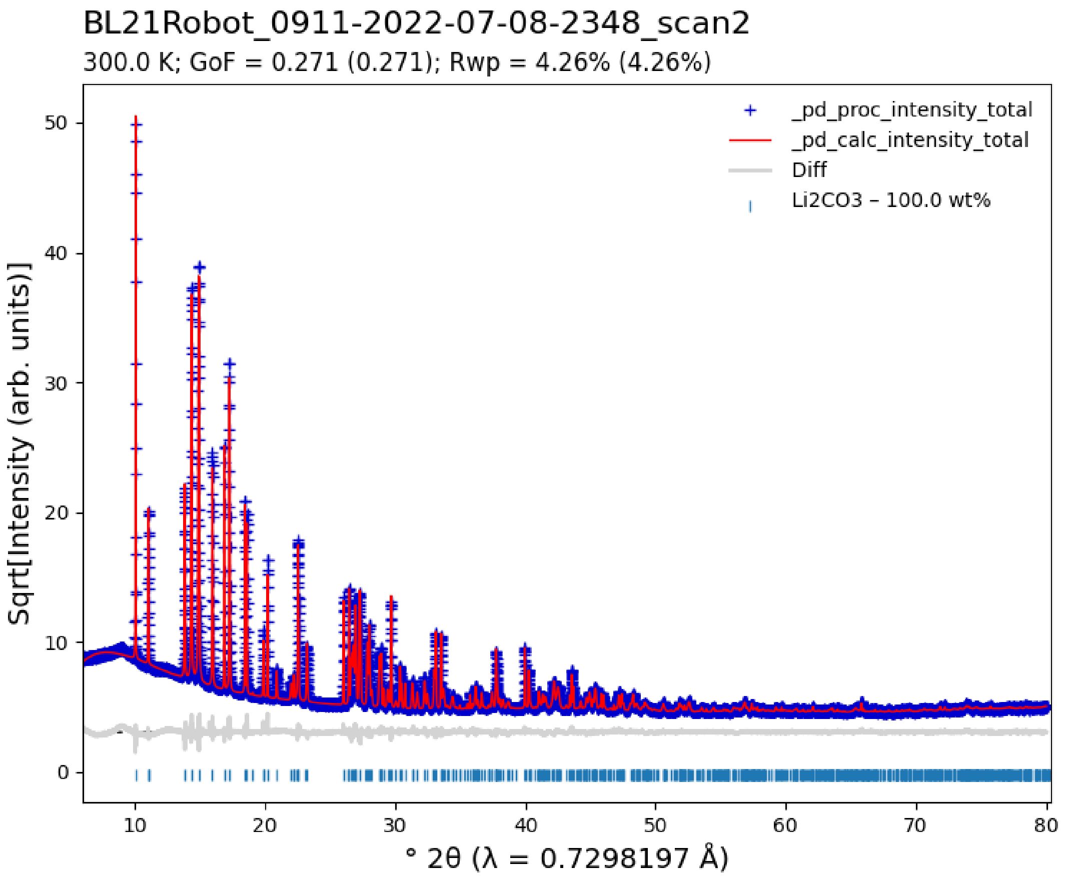

Figure S7 Rietveld refined data for sample BL21Robot_0911, lithium carbonate powder. Measured data are shown as blue crosses, calculated pattern in red with the difference shown below in grey. Tick marks indicate allowed peak positions.

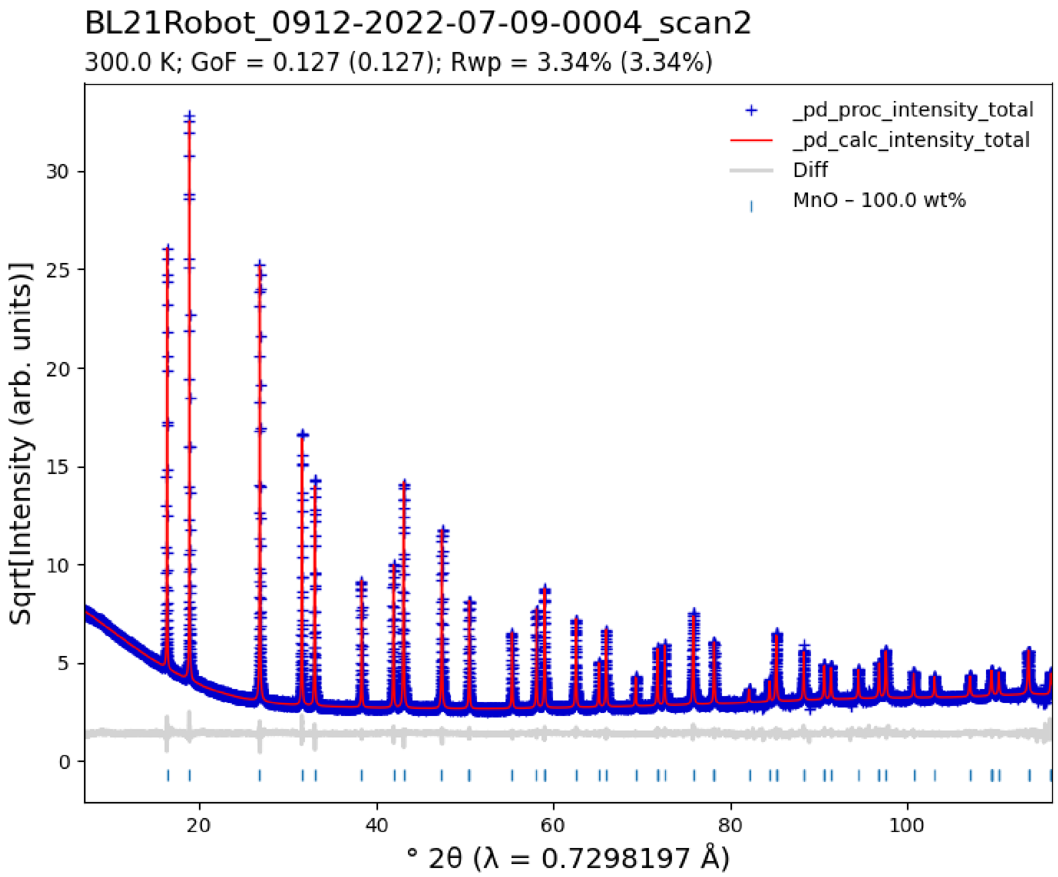

Figure S8 Rietveld refined data for sample BL21Robot_0912, manganese (II) oxide. Measured data are shown as blue crosses, calculated pattern in red with the difference shown below in grey. Tick marks indicate allowed peak positions.

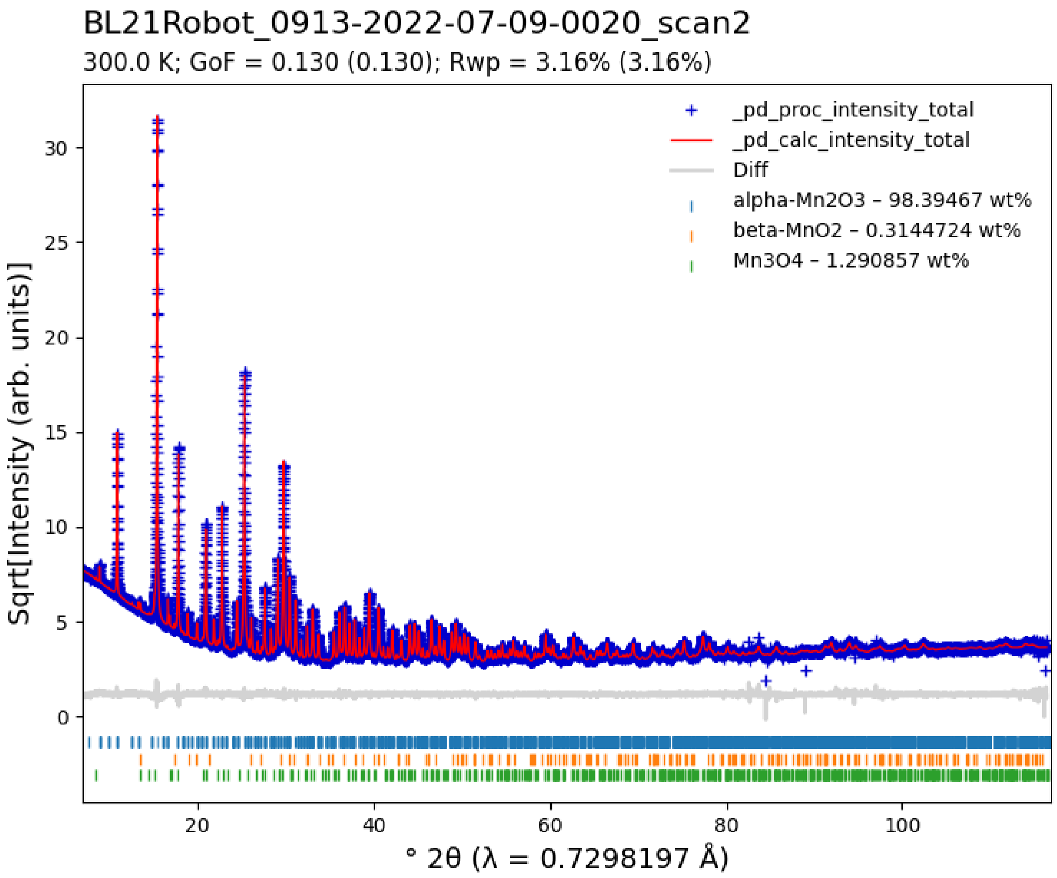

Figure S9 Rietveld refined data for sample BL21Robot_0913, manganese (III) oxide with minor impurities of β-MnO_2 and Mn_3O_4. Measured data are shown as blue crosses, calculated pattern in red with the difference shown below in grey. Tick marks indicate allowed peak positions.

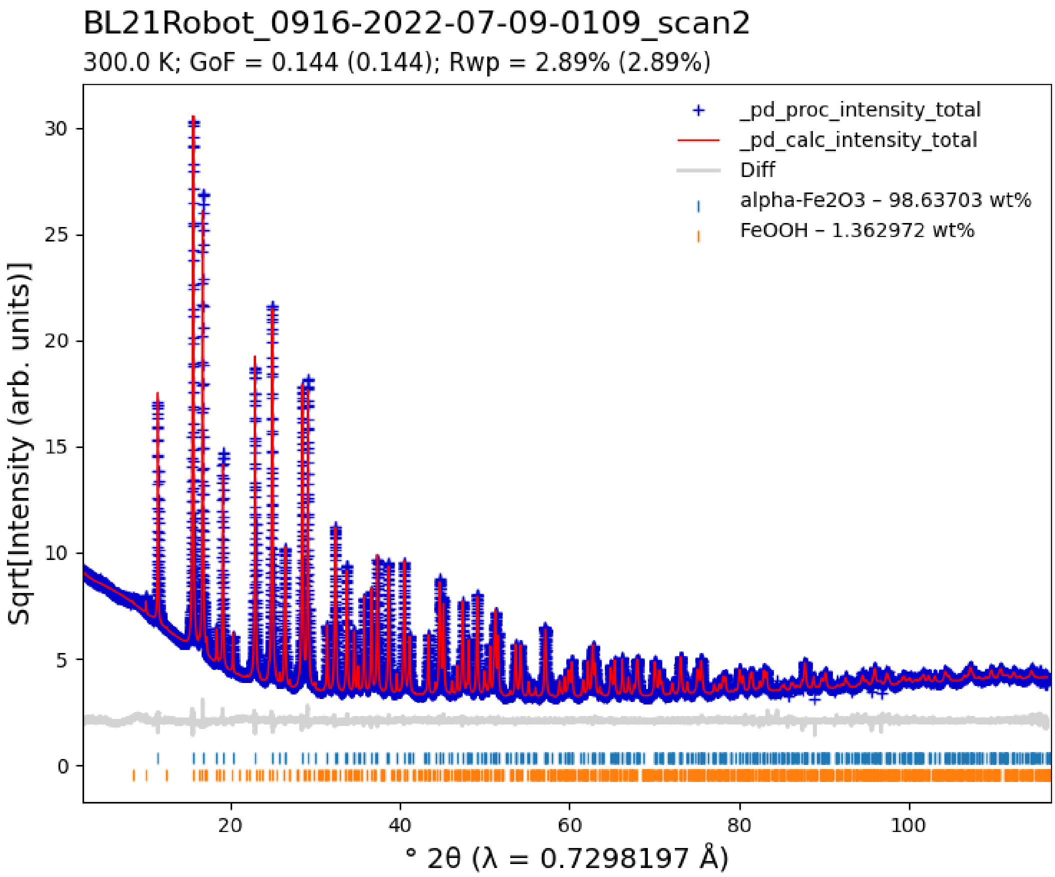

Figure S10 Rietveld refined data for sample BL21Robot_0916, iron (III) oxide with minor impurity of iron oxide-hydroxide. Measured data are shown as blue crosses, calculated pattern in red with the difference shown below in grey. Tick marks indicate allowed peak positions.

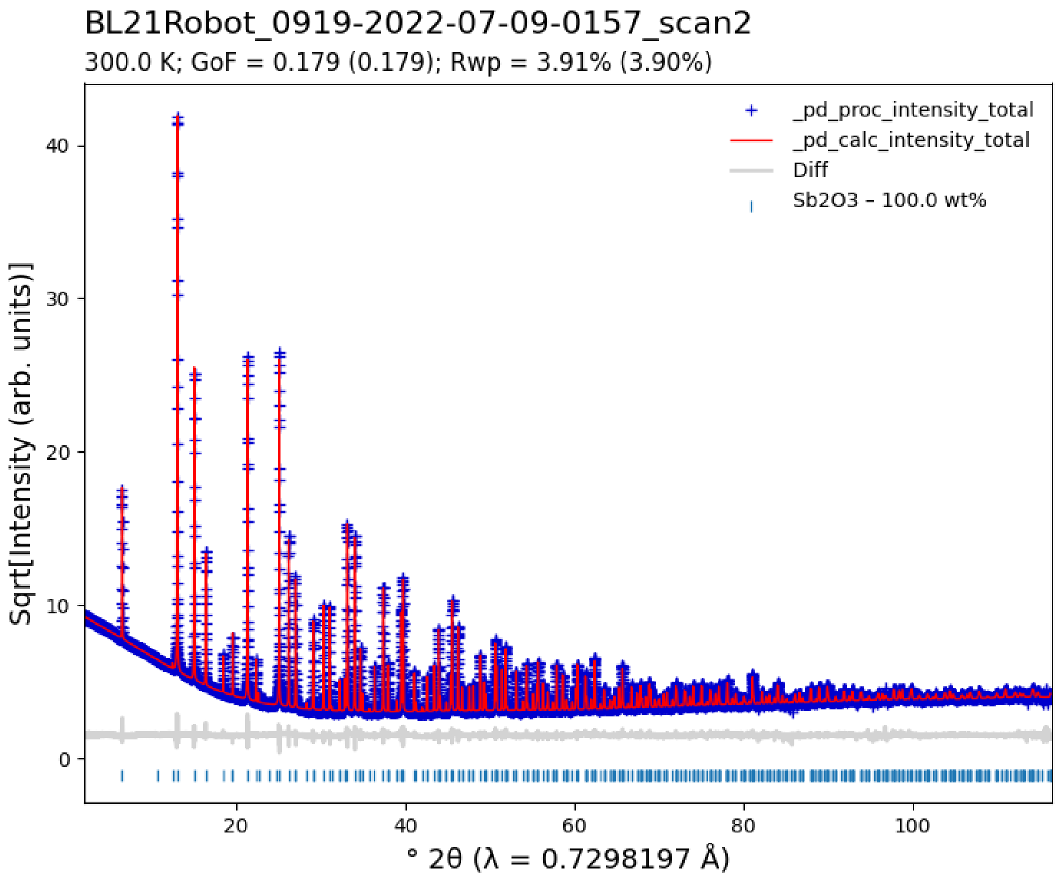

Figure S11 Rietveld refined data for sample BL21Robot_0919, antimony (III) oxide. Measured data are shown as blue crosses, calculated pattern in red with the difference shown below in grey. Tick marks indicate allowed peak positions.

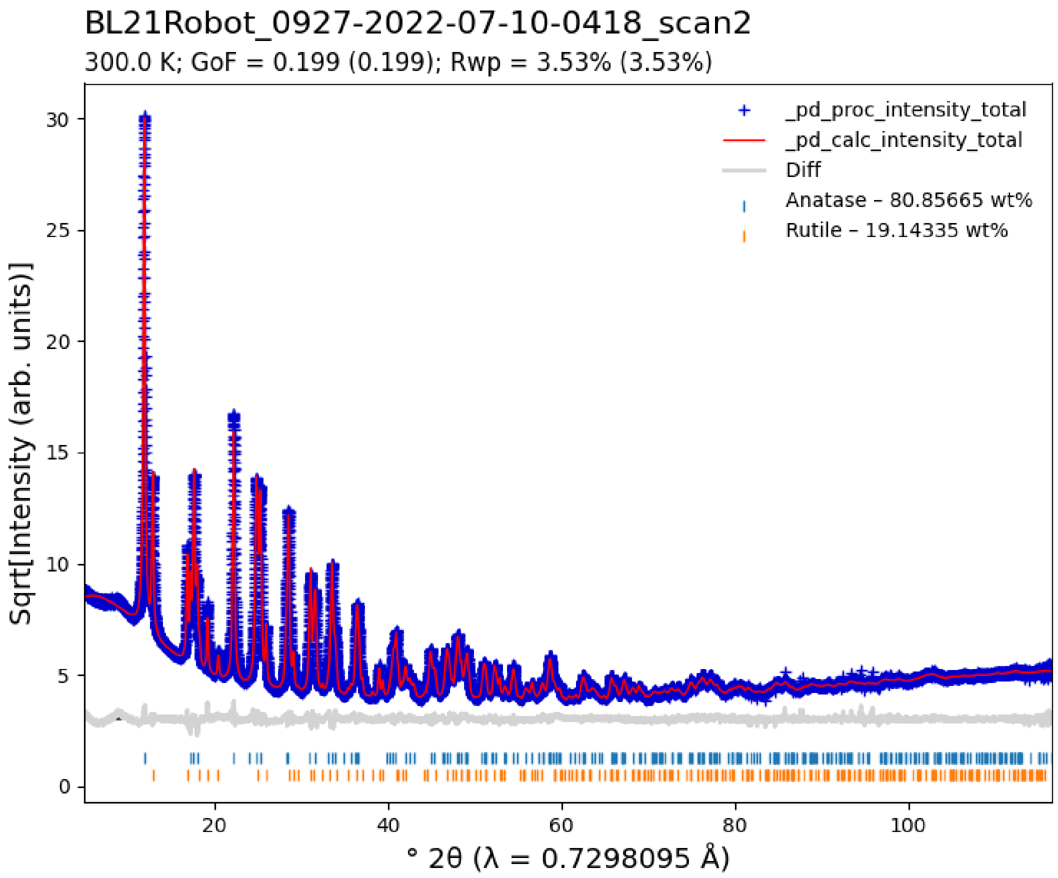

Figure S12 Rietveld refined data for sample BL21Robot_0927, titanium dioxide in both rutile and anatase phases. Measured data are shown as blue crosses, calculated pattern in red with the difference shown below in grey. Tick marks indicate allowed peak positions.

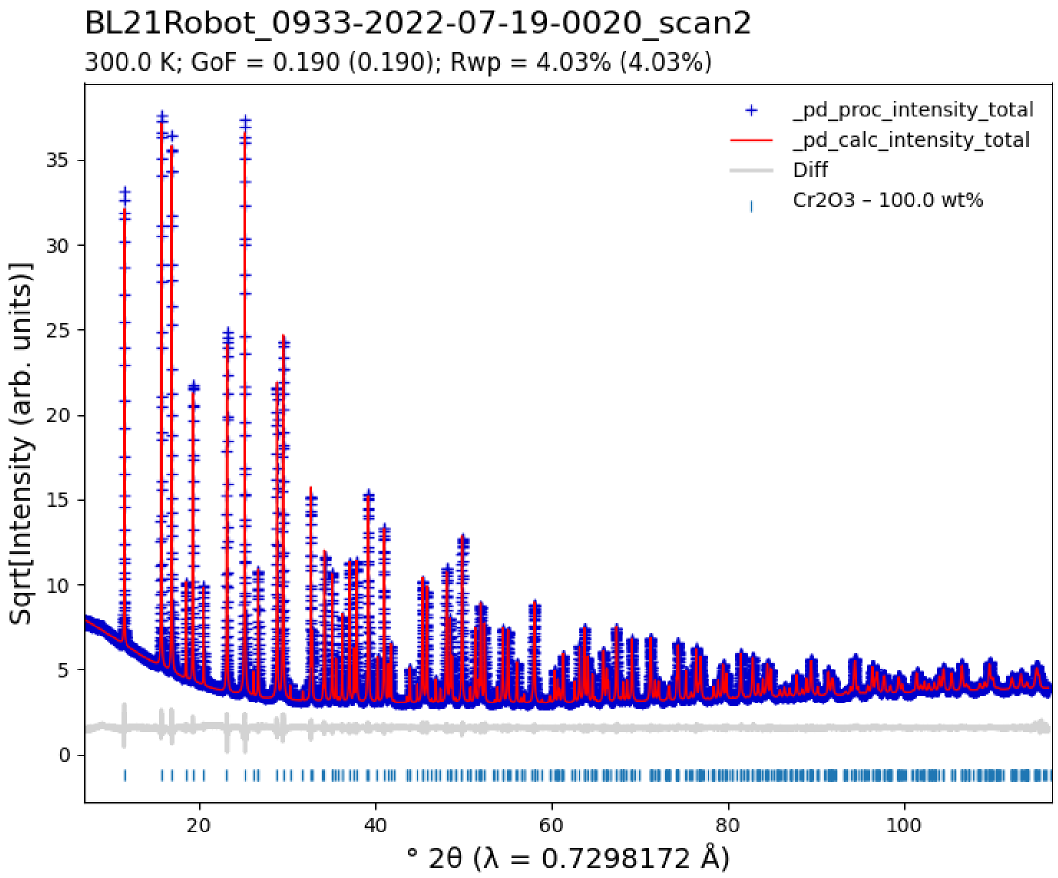

Figure S13 Rietveld refined data for sample BL21Robot_0933, chromium (III) oxide. Measured data are shown as blue crosses, calculated pattern in red with the difference shown below in grey. Tick marks indicate allowed peak positions.
